# Satisfaction, engagement, and outcomes in internet-delivered cognitive behaviour therapy adapted for people of diverse ethnocultural groups: an observational trial with benchmarking

**DOI:** 10.3389/fpsyt.2024.1270543

**Published:** 2024-03-04

**Authors:** Ram P. Sapkota, Emma Valli, Blake F. Dear, Nickolai Titov, Heather D. Hadjistavropoulos

**Affiliations:** ^1^ Online Therapy Unit, University of Regina, Regina, SK, Canada; ^2^ eCentreClinic, Macquarie University, Sydney, NSW, Australia; ^3^ MindSpot Clinic, eCentreClinic, Macquarie University, Sydney, NSW, Australia

**Keywords:** cognitive behaviour therapy, engagement, cultural adaptation, patient-oriented research, digital mental health, depression, anxiety, ICBT

## Abstract

**Introduction:**

Depression and anxiety are the most common mental health disorders worldwide. Internet-Delivered Cognitive Behaviour Therapy (ICBT) can reduce barriers to care to broad cross sections of the population. However, People of Diverse Ethnocultural Backgrounds (PDEGs) other than White/Caucasian underutilize mental health services and are under represented in clinical trials of psychological interventions.

**Methods:**

To address this research gap we adapted an evidence-based ICBT program for PDEGs. The current pilot study explores the engagement, satisfaction, and effectiveness in the adapted ICBT program by PDEGs (N=41) when benchmarked against a sample of PDEGs (N=134) who previously completed a non-adapted version of the ICBT program.

**Results:**

An intent-to-treat analyses showed that the adapted ICBT program is effective in reducing anxiety and depression symptoms among PDEGs. Large within-group pre-to post-treatment Cohen’s effect sizes of *d* = 1.23, 95% CI [0.68, 1.77] and *d* = 1.24, 95% CI [0.69, 1.79] were found for depression and anxiety, respectively. Further, 81.8% of the PDEGs who received the adapted ICBT reported overall satisfaction, 90.9% reported increased confidence in managing symptoms, and 70.7% completed majority of the psychoeducational lessons in the ICBT program.

**Conclusion:**

No statistically significant differences in the clinical outcomes, engagement, and satisfaction were found between the pilot study and benchmark sample. Future directions for ICBT research with PDEGs are described.

**Clinical trial registration:**

https://beta.clinicaltrials.gov/study/NCT05523492, identifier NCT05523492.

## Introduction

Depression and anxiety are among the most common mental health concerns worldwide (1). As is remarked often, with the availability of evidence-based digital mental health interventions such as transdiagnostic Internet-Delivered Cognitive Behaviour Therapy (ICBT) (e.g., 2, 3), help is just a click away. Yet a significant gap exists between those who have access to and use these services. For example, a recent analysis of ICBT utilization trends over six years (i.e., 2013-2019) in an online clinic in Saskatchewan, Canada showed that there has been a consistently lower (~10% compared to the expected population of ~32%) participation from self-identified non-White/Caucasian people in ICBT (4). People of Diverse Ethnocultural Backgrounds (PDEGs) other than White/Caucasian are known to underutilize mental health services (e.g., 5), and are also underrepresented in clinical trials of psychological interventions including ICBT (6, 7). Further, despite the recognition of the salience of ethnocultural backgrounds on mental health (e.g., 8, 9), there has been limited research on adapting ICBT to be appropriate for multiple ethnocultural groups with the aim of improving its utilization by PDEGs in routine care settings (10). Of note, for the lack of better terminology, we have used the phrase People of Diverse Ethnocultural Groups (PDEGs) to refer to all but self-identified Caucasian/White people in this study. In this regard, PDEGs encompass Indigenous Canadians as well as “visible minorities” or “racialized minorities” such as black, Asian, and Latin American. Other terms for this group that are used in the literature include visible monitories, racialized minorities, or Black, Indigenous, People of Colour (BIPOC).

To address this research gap as well as with an aim to increase use of ICBT by PDEGs in an online routine care clinic in Saskatchewan, Canada, we conducted a multi-phase patient-oriented research [see (11)] project and adapted an evidence-based ICBT program based on feedback from self-identified PDEG patients, community representatives providing services to PDEGs, and ICBT clinicians [see (12) for details of the adaptation process]. Suggestions for improvement including acknowledging the role of culture on mental health and mental health stigma in the course materials, diversifying case stories, examples and images, language simplification and audiovisual materials but did not involve modifying the cognitive behavioural approach. Moreover, further trainings were offered to clinicians and efforts were made to improve community outreach (see 12). This approach to adaptation is aligned with the Selective and Directed Treatment Adaptation Framework [SDTAF; (13, 14)], which has strong emerging evidence base to guide adaptations of Western psychological interventions for other populations. The current pilot study was conducted to assess the effectiveness, satisfaction, and engagement of the adapted ICBT by PDEGs benchmarked against data from PDEGs who took a non-adapted version of the same ICBT program in the past. Given the changes made, we expected similar effect sizes, but increased engagement and satisfaction with the adapted ICBT when benchmarked against a sample of PDEGs who took a non-adapted version of the ICBT program (see 15).

## Materials and methods

### Participants

**Figure 1:**
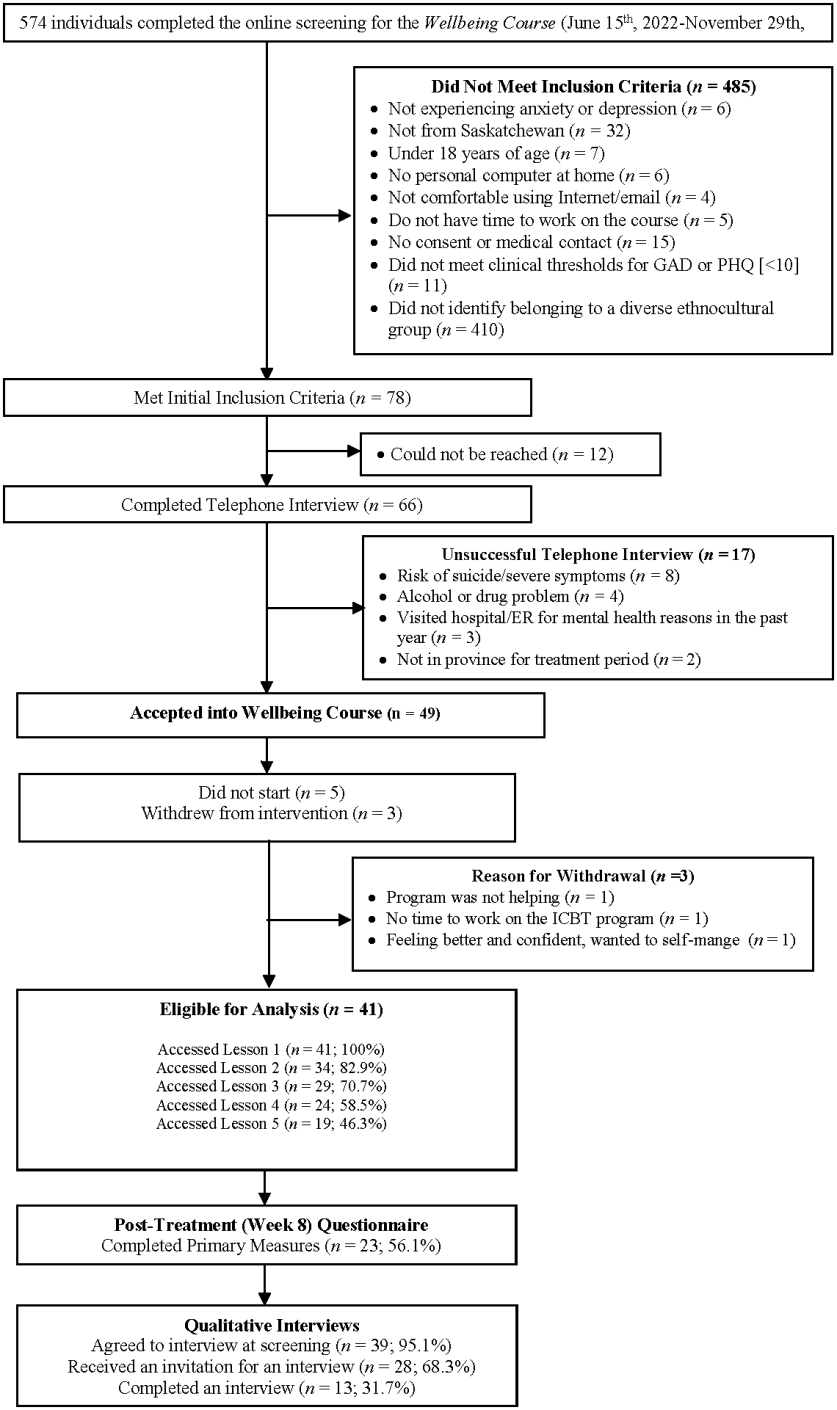
Participant flow diagram.

This registered pilot interventional trial (NCT05523492; https://beta.clinicaltrials.gov/study/NCT05523492) used a single-group pre- to post-treatment design. Prospective participants learned about the Online Therapy Unit (www.onlinetherapyuser.ca) through a variety of sources, such as family physicians, other medical professionals, community mental health clinics, web-based searches, word of mouth, media, and posters or cards. To access the course, participants completed an online questionnaire followed by a telephone interview. During this interview, staff determined if participants met basic eligibility for the Course (i.e., ≥18 years of age, Saskatchewan resident, endorsed depression and/or anxiety symptoms, had access to a computer and the Internet, able to provide a healthcare provider emergency contact), or were experiencing concerns outside of the scope of the Course (i.e., are at a high risk for suicide, have significant alcohol and/or drug usage, or are experiencing unmanaged psychosis or mania). One additional inclusion criterion specific to this study was that patients had to self-identify as belonging to PDEGs. Participants who did not meet the eligibility criteria were directed to more appropriate local services (see 4for further details). All eligible PDEG participants (N=41) enrolled in ICBT at the Online Therapy Unit between June 2022 and November 2022 were included (see **Figure 1**). Sub-sample (e.g., self-identified non-White/Caucasian) data (N=134) collected between December 2021 and May 2022 at the same site with an equivalent protocol were used as the benchmarking sample [see (15) for details about benchmarking sample].

At post-treatment ~32% (n=13) consenting participants in the pilot study were interviewed asking about their expectations and experience with the adapted ICBT Wellbeing Course and suggestions for further improvement.

### ICBT program: The Wellbeing Course

Participants were enrolled in the Adapted Wellbeing Course. This transdiagnostic course originally developed by Titov, Dear (16) addresses both depression and anxiety symptoms and was adapted as described above for PDEGs. The Course includes five online psychoeducational lessons and described (a) the cognitive-behavioural model and the relationship between thoughts, behaviours and emotions; (b) thought monitoring and challenging; (c) de-arousal strategies and pleasant activity planning; (d) graded exposure and behavioural activation; and (e) relapse prevention. This course is completed over ~ 8 weeks and complemented with once-weekly therapist emails or telephone calls where therapists answer patients’ ‘ questions about the course and provide guidance on use of skills. Each lesson includes a brief video, educational materials, case stories, homework suggestions (i.e., do-it-yourself guides) and frequently asked questions along with additional reading if patients are interested. Weekly automated email reminders are sent to remind patients’ of the Course [see (16) for details].

### Measures

Data were self-reported. Demographic variables (e.g., age, sex, ethnicity, education, marital status, employment status, etc.) were recorded at pre-treatment. Considering vast diversity in ethnocultural backgrounds of patients in the Canadian context (17), ethnicity was assessed using broadly used ethno-racial categories, that is, black, white, Asian (east, middle east, south), Indigenous (First Nations, Inuit, Métis), Latin American, and other (with the option for participants to describe their ethnicity).

Consistent with past research (4, 18), ICBT engagement was assessed using number of patient messages, phone calls, and lessons accessed. Following previous research, for treatment satisfaction, at post-treatment, participants answered yes/no items about whether they would recommend the treatment to a friend, and if the course was worth their time. Participants also rated satisfaction with the program, materials, and confidence to manage symptoms, and motivation to seek future treatment if needed (1 to 5 scale). Patients completed measures of depression [Patient Health Questionnaire-9 [PHQ-9]; (19)] and anxiety [Generalized Anxiety Disorder-7 [GAD-7]; (20)] at pre-treatment (baseline), each week for seven weeks, and post-treatment (end of week 8). Scores ≥10 in these measures indicate clinically significant symptoms of depression or generalized anxiety. Cronbach’s α in this study was between.66 and.91, and.81 and.93 for PHQ-9 and GAD-7, respectively.

The post-treatment qualitative interviews included 12 open-ended questions that asked about the patient’s experience with the program, including perceived cultural relevance of different aspects of the ICBT program, and ways to improve accessibility and utilization of the program for PDEGs [see (12) for details].

### Data analysis

Statistical analyses were conducted using IBM SPSS Statistics (Version 28.0). Descriptive statistics described patients’ characteristics. Pilot versus benchmark groups were compared on pre-treatment characteristics, engagement and satisfaction measures using Chi square tests for categorical variables and t-tests for continuous variables. A series of mixed model analyses were run using all data available across the nine weekly assessments to examine changes in the PHQ-9 and GAD-7 outcomes over time and to determine if these changes differed between groups. For each outcome, a series of models involving fixed and random effects of intercept and slope (time) were conducted. The fixed-effect models included time, group, and their interaction (time × group). Intraclass correlation coefficients were used to determine if mixed-model analyses were appropriate (21). Various within-individual covariance structures (e.g., scaled identity, diagonal, autoregressive [AR (1)]) were also tested. The models with smallest Akaike’s Information Criterion and Bayesian Information Criterion were selected for the final analysis. Estimates were calculated using the full information maximum likelihood method. These analyses were conducted twice, 1) using an intention-to-treat (ITT) sample, for which data were imputed using the Multiple Imputation method such that all enrolled participants were included, and 2) using a complete case analysis, for which unimputed data were used. For the ITT analyses, twenty multiply imputed data sets were created (22). Since mixed-model analysis with maximum likelihood method of estimation can handle missing data, imputation prior to analysis was not necessary for complete case analysis (23, 24). Pre- to post-treatment effect sizes (Cohen’s *d*) were computed using estimated means and standard deviations from the mixed model analysis. Further, we compared the groups on reliable change by calculating the percentage of participants who recovered (GAD-7 or PHQ-9 ≤ 4), improved reliably (GAD-7 or PHQ-9 ≤ 9), or did not show reliable improvement (GAD-7 or PHQ-9 ≥ 10). Pre-treatment standard deviations and test-retest reliability estimates α = .84 for PHQ-9 (19) and α = .83 for GAD-7 (20) were used to compute reliable change index (25).

The digitally recorded post-treatment qualitative interviews were transcribed verbatim, coded by combining deductive and inductive approaches, and organised according to themes for thematic analysis (26). The frequency of each code was recorded for descriptive analysis. The semi-structured interview data were coded by a researcher (EV) using NVivo (version 12 Plus) software.

## Results

A total of 41 participants from the pilot study (see **Figure 1** for the participant flow chart) and 134 participants from the benchmarking study were included (see **Table 1** for pre-treatment demographic and clinical characteristics for the groups). No group differences were found (see **Table 1**). Participants were mostly Indigenous (53.1%), women (74.9%), married (50.3%), with an average age of 32.9 years (SD = 10.6 years) residing in large cities (64%).

**Table 1:** Patients’ pre-treatment characteristics by group.

Pre-treatment characteristics	All participants (N=175)	Pilot study (N=41)	Benchmark study (N=134)	Significance *(X* ^2^ or *t*-test)
	n (%)	n (%)	n (%)	
Age				*p*=.328
Mean (SD)	32.99 (10.64)	31.56 (10.99)	33.43 (10.5)	
Gender				*p*=.060
Women	131 (74.9)	26 (63.4)	105 (78.4)	
Men	41 (23.4)	13 (31.7)	28 (20.9)	
Others (Non-binary & transgender)	3(1.7)	2 (4.9)	1 (0.7)	
Education				*p*=.308
High school diploma or less	48 (27.4)	15 (36.6)	33 (24.6)	
Post high school certificate/diploma	72 (41.1)	14 (34.1)	58 (43.3)	
University education	55 (31.4)	12(29.3)	43(32.1)	
Employment status				*p*=.532
Employed	77 (44.0)	22 (53.7)	55 (41.0)	
Unemployed	19 (10.9)	4 (9.8)	15 (11.2)	
Disability	23 (13.1)	5 (12.2)	18 (13.4)	
Others (e.g., homemaker, student, retired)	56 (32.0)	10 (24.4)	46 (34.3)	
Marital status				*p*=.557
Single, never married, dating	74 (42.3)	20 (48.8)	54 (40.3)	
Married, common law, living with a partner	88 (50.3)	19 (46.3)	69 (51.5)	
Separated, divorced, widowed	13 (7.4)	2 (4.9)	11 (8.2)	
Ethnicity				*p*=.607
Asian (East, West & South)	47 (26.9)	13 (31.7)	34 (25.4)	
Black	13 (7.4)	3 (7.3)	10 (7.5)	
Indigenous (First Nations, Inuit, Métis)	93 (53.1)	22 (53.7	71 (53.0)	
Latin American	7 (4.0)	0(0.0)	7 (5.2)	
Others	15 (8.6)	3 (7.3)	12 (9.0)	
Location				*p*=.879
Small rural location	42 (24.0)	10 (24.4)	32 (23.9)	
Small to medium city	21 (12.0)	4 (9.8)	17 (12.7)	
Large city	112 (64.0)	27 (65.9)	85 (63.4)	
Mental health characteristics				
Depression; Mean (SD)	16.26 (5.19)	16.59 (4.25)	16.16 (5.46)	*p*=.606
Anxiety; Mean (SD)	15.07 (4.15)	15.41 (4.29)	14.97 (4.12)	*p*=.550

For both groups, there were no missing data across pre-treatment demographic and clinical variables but there were 55 (41.0%) and 17 (41.5%) missing values in the benchmark and pilot samples, respectively, at post-treatment. In both groups, data were missing mainly due to dropout. There were no statistically significant differences in pre-treatment demographics, and anxiety and depression symptom severity between the dropouts and completers, therefore the data were assumed to be missing at random (27).

### Engagement

There were no significant differences in attrition between pilot (41.5%) and benchmark (41.0%) groups on post-treatment outcome variables (*p* = .55) or mean number of lessons completed (pilot: 3.78 (SD = 1.79) vs benchmark: 3.99 (SD = 1.74), *p* = .47). Also, the groups did not significantly differ on the mean number of messages and phone calls to therapists (see **Table 2**).

**Table 2:** Engagement, treatment satisfaction and negative effects by group.

Variables	All participants n (%)	Pilot study sample n (%)	Benchmarking study sample n (%)	Significance *(X^2^* or *t*-test)
Engagement	(N= 175)	(N=41)	(N=134)	
Completed ≥ 3 lessons	123 (70.3)	29 (70.7)	94 (70.1)	*p*=.943
Completed all lessons	91 (52.0)	19 (46.3)	72 (53.7)	*p*=.407
Message sent to therapists; Mean (SD)	7.21 (2.35)	7.02 (2.44)	7.27 (2.33)	*p*=.562
Number of phone calls; Mean (SD)	1.22 (1.29)	1.22 (1.13)	1.22 (1.33)	*p*=.989
Satisfaction	(N=101)	(N=22)	(N=79)	
Satisfied overall	77 (76.2)	18 (81.8)	59 (74.7)	*p*=.487
Satisfied with materials	84 (83.2)	20 (90.9)	64 (81.0)	*p*=.350*
Increased confidence to manage their symptoms	83 (79.7)	20 (90.9)	63 (79.7)	*p*=.347*
Increased motivation to seek other treatments if needed	87 (86.1)	20 (90.9)	67 (84.8)	*p*=.729*
Course was worth the time	95 (94.1)	21 (95.5)	74 (93.7)	*p*=1.00*
Would confidently recommend the course to a friend	77 (97.5)	21 (95.5)	98 (97.0)	*p*=.525*
Negative effects	(N=100)	(N=22)	(N=78)	
Reported negative effects	11 (11.0)	1(4.5)	10 (12.8)	*p*=.448*

SD, Standard deviation; *Fisher’s Exact Test.

### Satisfaction

Although slightly higher percentage of pilot study participants reported satisfaction in most of the variables, there was no statistically significant differences between pilot and benchmark groups on satisfaction variables (see **Table 2**).

### Effectiveness

**Figure 2:**
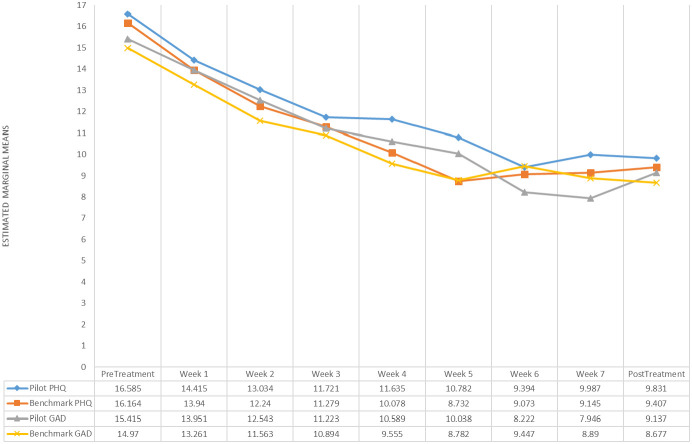
Weekly change in PHQ-9 and GAD-7 mean scores.

As expected, ITT analysis yielded a non-significant interaction effect for the PHQ-9 (*β*_1_ = − 0.01, 95% *CI* = [− 0.28, 0.21], *p* = .943) and the GAD-7 (*β*_1_ = − 0.13, 95% *CI* = [− 0.35, 0.09], *p* = .261). Similar results were obtained for the complete case analysis of the PHQ-9 (*β*_1_ = − 0.03, 95% *CI* = [− 0.29, 0.34], *p* = .866) and GAD-7 (*β*_1_ = -0.09, 95% *CI* = [− 0.41, 0.22], *p* = .553), suggesting that the change in self-reported depression and generalized anxiety symptoms over 9 weeks was not significantly different between groups (see **Figure 2**). For both groups, significant reductions on the PHQ-9 (*β*_1_ = − 0.82, 95% *CI* = [− 1.01, − 0.64], *p* < .001) and GAD-7 (*β*_1_ = − 0.79, 95% *CI* = [− 0.94, − 0.65], *p* < .001) were observed over time in ITT analyses as well as in the complete case analyses, PHQ-9 (*β*_1_ = − 0.91, 95% *CI* = [− 1.06, − 0.57], *p* < .001) and GAD-7 (*β*_1_ = − 0.80, 95% *CI* = [− 0.95, − 0.64], *p* < .001). Final models used AR (1) within-individual covariance structure. Similar large pre-to-post within-group effect sizes (Cohen’s d) were found for the PHQ-9 in the pilot (*d* = 1.23, 95% CI [0.68, 1.77]) and benchmark (*d* = 1.18, 95% CI [0.88, 1.48]) groups and for the GAD-7 pilot (*d* = 1.24, 95% CI [0.69, 1.79]) and benchmark (*d* = 1.27, 95% CI [0.97, 1.58]) groups.

### Clinically significant change

On the PHQ-9 at post-treatment, for the pilot group (n=24), 10 (41.7%), 4 (16.7%), and 10 (41.7%) met criteria for recovered, reliable improvement, and did not improve reliably, respectively. In the benchmark group (n=79), 27 (34.2%), 19 (24.1%), and 33 (41.8%), met criteria for recovered, reliable improvement, and did not improve reliably, respectively, on the PHQ-9. Similarly, on the GAD-7, in the pilot group (n=24), 8 (33.3%), 6 (25.0%), and 10 (41.7%) met criteria for recovered, reliable improvement, and did not improve reliably, respectively. In the benchmark group (n=79), 28 (35.4%), 20 (25.3%), and 31 (39.2%) met criteria for recovered, reliable improvement, and did not improve reliably, respectively.

### Negative effects

One (2.4%) pilot study participant and 11 (8.2%) benchmark study participants self-reported negative effects during the treatment period. In both groups, negative effects were related to transient increases in symptoms during ICBT, such as, when confronting bad memories, when challenging thoughts, or thinking about symptoms.

### Post-treatment interviews

Thirteen self-identified PDEG participants representing Indigenous (53.8%), Asian (23.1%) and Black (23.1%) communities were interviewed. There were equal number of self-identified men and women (n=6, 46.2% each) and one (7.7%) transgender participants, with an average age of 31 years (SD = 11.4 years).

A majority of the interview participants (n=12, 92.3%) indicated that the ICBT program met or exceeded their expectations and reported having positive experiences with the therapists. Likewise, in terms of cultural fit of the ICBT program, a majority (n=10, 76.9%) said that the program aligns with their cultural beliefs and practices. When asked for suggestions for specific improvements to make the program better for people from their ethnocultural background, seven (53.8%) participants mentioned that there was no need for change in the program. The remaining (n=6, 46.2%) participants provided some suggestions for further improvements: making the public more aware about this freely available program (n=2, 15.4%), adding culture-specific content (n=2, 15.4%), diversifying the cultural background of the main characters represented in the case stories (n=2, 15.4%), language translation (n=1, 7.7%), and increasing therapist support (n=1, 7.7%).

## Discussion

We examined the treatment engagement, satisfaction, and outcomes of an ICBT program adapted for PDEGs benchmarked against a standard (non-adapted) ICBT program. The results showed that there were no statistically significant differences in the engagement, satisfaction, and outcomes between the pilot and benchmark samples, which may reflect the sample size and comparison of the adapted ICBT program to an already established and effective ICBT program. It is unclear at this point whether further adaptations to the ICBT program would serve to enhance the engagement, satisfaction, and outcomes above and beyond the standard ICBT program. Interviews revealed patients were largely satisfied with the adapted program and there was little consensus on suggestions for improvement suggesting further adaptations may not result in significant benefit and the current outcomes reflect the maximal benefits with this line of treatment.

While statistically significant differences were not observed, some non-measured benefits have yet to be examined, including whether offering this adapted ICBT course will improve uptake of the course by PDEGs. Furthermore, although this study did not provide strong evidence that patient-oriented adaptation of the ICBT improves clinical outcomes, engaging PDEG patients and other groups who work with PDEGS has provided some assurance that the adapted ICBT program is culturally relevant and clinically significant in reducing symptoms of anxiety and depression across diverse ethnocultural groups. In the future, we intend to explore whether there is an increased uptake of ICBT by PDEGs. However, we acknowledge that underutilization of ICBT could reflect increased resilience, differences in help-seeking behaviour, and that PDEGs may prefer alternative sources of support.

## Limitations

This study had several limitations. We could not conduct more detailed ethnocultural subgroup analyses (e.g., Asian group includes many diverse ethnicities and cultures) due to small sample size in our pilot study and the lack of data on place of birth, immigration history, level of acculturation etc., past research (e.g., 28, 29) shows that younger adults tend to engage less and drop-out more from the ICBT program. Further, there is some evidence that younger immigrants likely acculturate faster (see 30, 31) and more acculturated people are less likely to benefit from culturally adapted interventions than less acculturated people (32). In the current study, the mean age of the participants was 31.6 (SD=10.9) (see **Table 1**), which was lower than in our previous studies (Mean age = 38.14, SD = 11.9 years) (see 4). Further, consistent with the literature, engagement was measured using number of lessons completed and email and/or telephone exchange with therapists. However, although these measures are useful in assessing behavioural aspects of engagement, they do not necessarily reflect the level of engagement (e.g., effort put into reviewing the lessons, practicing skills, continuity in use of the learned skills) with the program. All these factors may have influenced that rate of engagement and effectiveness of the adapted intervention in the current study. Further studies should consider involving more diverse range of age groups and more nuanced measures of engagement (see 33).

Within a setting that includes PDEGs, limited organizational resources do not allow for development of ICBT for each ethnocultural group accessing services. Nevertheless, adaptation to specific larger groups could be helpful such as Indigenous peoples using Indigenous research methods as has been done in Australia (see 34). Meta-analytic evidence suggests that interventions targeted to specific ethnocultural group are more effective than interventions adapted for multicultural settings (32). In the case of internet- and mobile-based interventions for mental health disorders, however, there is a dearth of research that directly compares adapted and non-adapted interventions (35) and more research is needed to understand the benefits of adaptation and which approaches are most effective. Of note, non-adapted ICBT interventions have also been demonstrated to produce equivalent outcomes in migrants and non-migrants in Australia (36) and elsewhere (see 37). We opted for a patient-oriented adaptation as it was not feasible (based on available resources) for us to conduct a comprehensive cultural adaptation of the ICBT program considering ICBT is being implemented in a multicultural routine care setting in Saskatchewan. Therefore, a thought for future studies remains, that is, as the existing research suggests (e.g., 38), perhaps we would obtain better engagement, satisfaction, and treatment outcomes had the ICBT program been culturally adapted and tailored to specific ethnocultural groups. Therefore, the findings of this study are preliminary and should not diminish the importance of need-informed cultural adaption, especially targeting a specific ethnocultural group in the context of digital mental health. Adaptation of therapeutic programs is indeed a complex issue that calls for careful consideration. As noted in a recent review of the literature on the effectiveness of adapted psychological interventions, various types of cultural adaptations (e.g., adaptation of content, method of delivery, training of therapists, etc.) may have different effects on outcomes. Additionally, the review suggests that some ethnocultural groups may benefit more from cultural adaptation than others [e.g., South Asians may benefit more than East Asians; (39)].

## Data availability statement

The raw data supporting the conclusions of this article will be made available by the authors, without undue reservation.

## Ethics statement

All procedures contributing to this work comply with the ethical standards of the relevant national and institutional committees on human experimentation and with the Helsinki Declaration of 1975, as revised in 2008. The studies involving humans were approved by the Institutional Review Board of the University of Regina (benchmark study: REB # 2019-197, December 12, 2019; pilot trial: REB # 2022-012, March 17, 2022). The studies were conducted in accordance with the local legislation and institutional requirements. The participants provided their written informed consent to participate in this study.

## Author contributions

RS: Conceptualization, Data curation, Formal analysis, Funding acquisition, Investigation, Methodology, Validation, Visualization, Writing – original draft, Writing – review & editing. EV: Investigation, Project administration, Writing – original draft, Writing – review & editing. BD: Investigation, Validation, Writing – review & editing. NT: Investigation, Validation, Writing – review & editing. HH: Conceptualization, Funding acquisition, Investigation, Methodology, Project administration, Resources, Validation, Writing – original draft, Writing – review & editing.
